# Clinical Course and Conservative Strategy for Persistent De Novo Stress Urinary Incontinence After Pelvic Organ Prolapse Repair with Transvaginal Mesh

**DOI:** 10.3390/biomedicines13081975

**Published:** 2025-08-14

**Authors:** Yu-Ling Tu, Kun-Ling Lin, Zi-Xi Loo, Yao-Yu Yang, I-Chieh Sung, Cheng-Yu Long

**Affiliations:** 1 Department of Obstetrics and Gynecology, Kaohsiung Medical University Hospital, Kaohsiung Medical University, Kaohsiung 80708, Taiwan; patty831017@gmail.com (Y.-L.T.); nancylin95@gmail.com (K.-L.L.); loozixi@hotmail.com (Z.-X.L.); 2 Department of Obstetrics and Gynecology, Gangshan Hospital of Kaohsiung City, Kaohsiung Medical University, Kaohsiung 80708, Taiwan; yaoyu1107@gmail.com (Y.-Y.Y.); starrynight278@gmail.com (I.-C.S.)

**Keywords:** de novo stress urinary incontinence, transvaginal mesh surgery, pelvic organ prolapsed, urinary continence, vaginal laser therapy

## Abstract

**Background/Objectives**: De novo stress urinary incontinence (SUI) can develop postoperatively in patients without prior symptoms, and can persist beyond 6 months, posing clinical challenges. This study aimed to identify predictors of persistent de novo SUI after transvaginal mesh (TVM) surgery and to evaluate management strategies. **Methods**: A retrospective review of 817 women with anterior and apical pelvic organ prolapse (POP) (stage II–IV) who underwent TVM surgery from 2013 to 2021 was conducted. Fifty patients developed de novo SUI postoperatively. Assessments included urodynamic studies, validated symptom questionnaires, and POP quantification (POP-Q) staging. Logistic regression analysis was used to identify predictors of persistent symptoms. **Results**: Spontaneous resolution occurred in 30% (15/50) of participants within six months, while 70% (35/50) had persistent SUI. Concomitant posterior mesh repair was more frequent in the persistent group compared to the self-limiting group (29% vs. 7%), and was significantly associated with symptom persistence (OR 5.6, 95% CI, 0.65–48.4; *p* = 0.03, chi-square test). During conservative management with observation alone, 30% (15/50) experienced spontaneous resolution within 6 months, while 70% (35/50) had persistent symptoms. Among those with persistent symptoms, 56% required no further treatment, 10% improved with vaginal laser therapy, and 4% underwent sling surgery. **Conclusions**: Conservative management remains critical in the early postoperative period, given the high rate of spontaneous symptom resolution. For persistent cases, minimally invasive options such as vaginal laser therapy may be beneficial. Notably, only 4% required anti-incontinence surgery.

## 1. Introduction

Pelvic organ prolapse (POP) affects up to 50% of women who have given birth, with an estimated 11% lifetime risk of requiring surgery for symptomatic POP [1]. Various mesh kits have been developed for POP surgery, and previous studies have shown that transvaginal mesh (TVM) is effective at providing good short-term results and with an acceptable complication rate [2,3].

Surgical correction for POP aims to restore anatomical support and improve quality of life. A notable postoperative complication after POP repairs is the development of de novo stress urinary incontinence (SUI) [4], which occurs in patients with no prior incontinence symptoms. This condition can significantly impact postoperative satisfaction and quality of life, often complicating the clinical course after POP surgery. The reported incidence of de novo SUI varies widely, with studies such as Cruz et al. and Long et al. reporting rates of 20.5% and 30%, respectively, at 12 months of follow-up [5,6]. Despite its frequency, the underlying pathophysiology of de novo SUI after POP repair remains incompletely understood.

While several studies have identified potential risk factors for de novo SUI, such as advanced age, previous history of SUI, and occult urodynamic stress incontinence, there is a paucity of data focusing specifically on the persistence of these symptoms beyond the early postoperative period.

In this study, we defined persistent de novo SUI as involuntary urine leakage that first appeared within the first month after TVM surgery in patients who had no preoperative SUI symptoms and persisted for more than six months postoperatively. Importantly, all patients who developed de novo SUI in our cohort reported the onset of symptoms within the first postoperative month. These symptoms were consistently identified and documented during routine follow up visits 1 and 3 months after surgery. This definition allows us to distinguish persistent de novo SUI from transient postoperative incontinence, which may occur shortly after surgery due to tissue healing or inflammation, but typically resolves within a few weeks or months [7,8].

Persistent de novo SUI poses a unique clinical challenge, requiring tailored management strategies to optimize patient outcomes. This study aims to identify predictors associated with persistent de novo SUI and to evaluate the effectiveness of conservative versus invasive treatment options in managing these patients. By clarifying these aspects, we hope to contribute to improved counseling, individualized treatment planning, and overall postoperative care for women undergoing TVM surgeries.

## 2. Materials and Methods

From November 2013 to September 2021, the medical records of women who underwent TVM surgery at a tertiary referral center in Taiwan were retrospectively reviewed. The surgical procedures utilized soft polypropylene mesh with the Prolift™, Perigee™, Uphold™, and Elevate™ transvaginal mesh systems [9,10,11,12]. A total of 817 women with stage 2 or greater anterior and/or apical compartment prolapse, classified using the POP quantification (POP-Q) system, were initially identified. Of these, 327 were excluded due to missing or incomplete postoperative urinary symptom dates, 210 had preoperative symptoms of stress or mixed urinary incontinence, 130 underwent concomitant anti-incontinence surgery, and 100 were lost to follow-up within six months postoperatively. After applying all exclusion criteria, 50 women who developed de novo SUI postoperatively, had no preoperative SUI symptoms, and did not undergo concurrent anti-incontinence procedures were included in the analysis.

We defined persistent de novo SUI as involuntary urine leakage that first appeared within the first month after TVM surgery in patients without any preoperative SUI symptoms, and persisted for more than six months postoperatively. All cases of de novo SUI were initially identified during routine follow-up visits at 1 and 3 months after surgery based on patient self-reports, which were documented by the attending urogynecologist. Urodynamic studies were performed pre- and postoperatively; however, the diagnosis of de novo SUI relied primarily on patient reported symptoms using a clinical stress test. This definition was used to distinguish persistent de novo SUI from transient postoperative incontinence.

All patients underwent comprehensive evaluations, including demographic data collection and a detailed medical history. Baseline demographic data included age, parity, body mass index (BMI), menopausal status, smoking history, underlying diseases, hysterectomy history, and documentation of any concomitant procedures. A thorough physical examination was performed and POP was classified according to the POP-Q system [13].

Assessments included preoperative and postoperative urodynamic studies (UDS) and both preoperative and postoperative validated symptom questionnaires. UDS—including uroflowmetry, filling and voiding cystometry, and urethral pressure profilometry—was conducted following the recommendations of the International Continence Society (ICS) guidelines, using a six-channel urodynamic monitor (MMS; UD2000, Enschede, The Netherlands) operated by an experienced technician.

Symptom severity and quality of life were assessed using validated instruments, including the Overactive Bladder Symptom Score (OABSS) [14], Urinary Distress Index (UDI-6) [15], Incontinence Impact Questionnaire (IIQ-7) [15], and International Consultation on Incontinence Questionnaire–Short Form (ICIQ-SF) [16].

Conservative management mainly consisted of clinical observation with routine follow-up. Some patients were advised on pelvic floor muscle exercises, but no standardized physiotherapy or pharmacotherapy was systematically applied due to variability in patient adherence and clinician practice.

### Statistical Analysis

Continuous variables were expressed as mean ± standard deviation or median. For group comparisons, the Student’s *t*-test was used for continuous variables with normal distribution, while the Mann−Whitney U test was applied for continuous variables without normal distribution. Categorical variables were compared using the Chi-square test. To identify factors associated with persistent de novo SUI, a two-tailed *p*-value of <0.05 was considered statistically significant.

## 3. Results

### 3.1. Clinical Outcomes

Among the 50 patients, 15 (30%) experienced spontaneous resolution of de novo stress urinary incontinence (SUI) within six months, whereas 35 (70%) had persistent SUI at the six-month follow-up.

### 3.2. Predictors of Persistent De Novo SUI

Comparative analysis between the self-resolving and persistent SUI groups identified the following:

- Significant predictor:
  Performance of concomitant posterior mesh repair (*p* < 0.05) (Table 1).
- Non-significant factors:
  Age
  Body mass index (BMI)
  Menopausal status
  Smoking history
  Medical comorbidities (Table 1).

### 3.3. Pelvic Organ Prolapse Quantification (POP-Q)

Preoperative and postoperative POP-Q values are summarized in Table 2. Anatomical correction of pelvic organ prolapse was successfully achieved in all patients following transvaginal mesh surgery.

### 3.4. Symptom and Quality of Life Assessments

Validated questionnaire scores, including the Urinary Distress Inventory-6 (UDI-6) and Incontinence Impact Questionnaire-7 (IIQ-7), demonstrated statistically significant improvements in the self-resolving SUI group compared to the persistent SUI group (*p* < 0.05) (Table 3).

### 3.5. Urodynamic Findings

Urodynamic changes are presented in Table 4. Postoperative evaluation revealed:

- A significant decrease in maximum urethral closure pressure (MUCP) levels only in the self-resolving SUI group (*p* < 0.05).
- No significant difference in postoperative MUCP levels between the two groups (*p* = 0.88).

## 4. Discussion

This study is the first to evaluate the predictors of persistent de novo stress urinary SUI following TVM surgery and to assess outcomes in patients with persistent symptoms. While previous research has focused on de novo SUI, most studies have not distinguished between transient and persistent cases, particularly beyond six months postoperatively. The factors associated with persistent de novo SUI in this extended timeframe remain underexplored.

Prior studies have identified various predictors for de novo SUI, including advanced age (over 64 years), higher BMI, severe POP-Q stage (>3), preexisting diabetes mellitus (DM), and low MUCP levels (<60 mmHg) [5,6,7]. However, these studies did not differentiate between transient and persistent SUI. In our study, the only significant predictor of persistent de novo SUI was concomitant posterior mesh repair.

Posterior mesh repair, commonly performed to address vaginal vault or posterior vaginal wall prolapse, can unintentionally affect pelvic floor dynamics. These surgical interventions may alter the natural tension and support mechanisms, potentially impacting urethral sphincter function and increasing the risk of urinary incontinence. Notably, studies have indicated that de novo POP in the anterior compartment is more likely to occur following apical and posterior prolapse repairs [17,18]. This suggests that posterior compartment repairs can inadvertently compromise anterior compartment support, thereby heightening the risk of stress urinary incontinence.

In contrast to prior studies [5,6,7,8], common demographic and clinical factors such as age, parity, BMI, menopausal status, preexisting hypertension, diabetes mellitus, and history of hysterectomy were not significant predictors of persistent de novo SUI in our study. These differences may be attributed to our study’s specific focus on persistent SUI and its longer follow-up period.

The self-resolving group exhibited significantly greater improvements in UDI-6 and IIQ-7 questionnaire scores, suggesting that symptom resolution significantly impacts patient well-being and quality of life. These findings emphasize the importance of distinguishing between temporary and persistent de novo SUI after surgery to better guide patient counseling and treatment strategies.

In our study, a significant postoperative decrease in MUCP was observed only in the self-resolving SUI group (*p* < 0.05), whereas no such change occurred in the persistent SUI group. Although this may appear counterintuitive—as reduced MUCP is typically associated with worsening incontinence—the observed decrease may instead reflect a physiological redistribution of urethral pressure following anatomical restoration of the pelvic floor after TVM surgery. Conversely, the absence of a significant MUCP change in the persistent SUI group suggests that symptom persistence may be driven by mechanisms beyond urethral closure pressure, such as altered pelvic floor dynamics or neuromuscular dysfunction. Additionally, the lack of a significant difference in postoperative MUCP between the two groups (*p* = 0.88) further supports the conclusion that MUCP alone is not a reliable predictor of persistent de novo SUI following POP surgery.

In our study, the rate of persistent de novo SUI at the six-month follow-up after TVM surgery was 70% (N = 35), while 30% (N = 15) of patients experienced spontaneous resolution of symptoms without any active intervention. These patients were managed conservatively with observation alone. The spontaneous resolution observed in 30% of cases highlights the importance of conservative management during the early postoperative period, as symptoms often resolve due to healing or inflammation.

Among the 35 patients with persistent SUI, symptom severity varied. The majority (56%; N = 28) experienced mild symptoms and required no further intervention. A smaller subset 10% (N = 5) reported improvement following vaginal laser therapy and only 4% (N = 2) ultimately required surgical correction. These findings underscore the need for a tailored management approach for persistent SUI. A stepwise strategy that progresses from conservative to invasive management enables urogynecologists to optimize treatment while minimizing unnecessary interventions.

Vaginal laser therapy emerged as an effective, minimally invasive option for managing persistent SUI, benefiting 10% (N = 5) of patients in the study. As a non-surgical approach, vaginal laser therapy offers an alternative for patients seeking relief without the risks associated with more invasive treatments.

The strengths of our study include its specific focus on persistent de novo SUI following TVM surgeries, a clearly defined follow-up period, and comprehensive evaluation of demographic, clinical, and urodynamic parameters. These findings provide valuable insights for personalized patient care, reinforcing the importance of a tailored, step-by-step management strategy to optimize treatment effectiveness and patient outcomes.

However, several limitations should be acknowledged in our study. First, the use of four different transvaginal mesh kits, although we did not record the number of patients treated with each specific type. All kits used were intended for anterior and/or apical pelvic organ prolapse repair, and existing literature suggests comparable functional and anatomical outcomes among these commonly used mesh types [19,20]. Therefore, we believe the variation in mesh kits is unlikely to have significantly influenced the overall results or conclusions of this study.

Second, the relatively small sample size and the lack of long-term follow-up beyond six months limit the generalizability of our findings. Multicenter studies with larger cohorts and extended follow-up periods are needed to validate these findings and explore additional factors influencing persistent SUI.

Third, in our cohort, conservative management strategies primarily consisted of clinical observation with scheduled follow-up visits, and, in some cases, simple advice on pelvic floor muscle exercises. No standardized physiotherapy protocols, pharmacotherapy, or adherence monitoring were implemented. Under this approach, 30% of patients achieved spontaneous resolution of persistent de novo SUI within six months, suggesting that a subset of cases may resolve without active intervention. However, the absence of standardized treatment and the variability in patient adherence likely contributed to heterogeneous outcomes. These limitations should be considered when interpreting the observed effectiveness of conservative management in this population.

## 5. Conclusions

In our study, conservative management proved effective during the early postoperative period, with 30% of patients experiencing spontaneous resolution of de novo SUI symptoms. Among those with persistent symptoms beyond six months, only 4% required additional anti-incontinence surgery. Posterior mesh repair was identified as a predictor of persistent de novo SUI, potentially due to altered pelvic support dynamics. Vaginal laser therapy appeared beneficial in a small subset of patients, although the limited sample size warrants caution in interpreting its efficacy. These findings underscore the value of a tailored, stepwise management strategy. Starting with observation and conservative measures before proceeding to invasive interventions may enhance patient outcomes while minimizing unnecessary procedures.

## Figures and Tables

**Table 1:** Demographic data are given as mean ± standard deviation or N (%).

	Self-Limiting (N = 15)	No Self-Limiting (Persistent SUI) (N = 35)	*p* Value
Mean age (years)	68.2 ± 8.1	68.2 ± 9.1	0.98 *
Mean parity	3.2 ± 1.2	2.8 ± 1.2	0.43 *
Mean BMI (kg/m^2^)	23.1 ± 2.8	24.2 ± 2.8	0.17 *
Menopause	15(100)	34(97)	0.44 **
Current smokers	0	0	
Diabetes Mellitus	1(7)	5(14)	0.16 *
Hypertension	9(60)	16(46)	0.31 *
History of hysterectomy	5(33)	7(20)	0.46 *
Concomitant procedures in the study			
Posterior mesh repair	1(7)	10(29)	0.03 *
Posterior repair	1(7)	6(17)	0.12 *
Vaginal hysterectomy	1(7)	1(3)	0.32 *

Follow up (months): 6–18 m, * Chi-square test, ** Student’s *t*-test.

**Table 2:** Pelvic organ prolapsed quantification (POP-Q) values before and after surgery. Data are given as median (range) or N (%).

	Self-Limiting (N = 15)			No Self-Limiting (N = 35)			Intergroup Pre-Op	Intergroup Post-Op
POP-Q Parameters (cm)	Pre-Op	Post-Op	*p* Value *	Pre-Op	Post-Op	*p* Value *	*p* Value *	*p* Value *
Aa	2.0 ± 1.1	−1.6 ± 1.3	0.005 *	1.6 ± 1.1	−1.6 ± 0.7	<0.001 *	0.63	0.75
Ba	4.2 ± 1.9	−1.5 ± 1.2	0.005 *	3.6 ± 2.8	−1.6 ± 0.7	<0.001 *	0.75	0.49
C	2.2 ± 3.6	−6.9 ± 1.9	0.005 *	2.1 ± 4.0	−7.5 ± 1.3	<0.001 *	0.73	0.54
Ap	−1.9 ± 1.6	−1.8 ± 0.6	0.34	−1.5 ± 1.7	−2.2 ± 0.7	0.057	0.53	0.54
Bp	2.3 ± 2.5	−1.8 ± 0.6	0.005 *	1.4 ± 3.7	−2.2 ± 0.7	<0.001 *	0.69	0.54
Tvl	9.3 ± 1.6	8.4 ± 1.2	0.026 *	9.1 ± 1.2	8.6 ± 1.2	0.079	0.65	0.57

Pre-op, preoperative; Post-op, postoperative; Tvl, total vaginal length. Wilcoxon signed rank test. * < 0.005.

**Table 3:** Changes in validated questionnaires before and after surgery. Data are given as median (range).

	Self-Limiting (N = 15)			No Self-Limiting (N = 35)			Intergroup Post-Op
	Pre-Op	Post-Op	*p* Value *	Pre-Op	Post-Op	*p* Value *	*p* Value *
OABSS	5.0(1–9)	4(0–8)	0.09	5(1–10)	4(0–10)	0.20	0.58
UDI-6	27.8 (0–66.7)	11.1 (5.6–33.3)	0.02	22.2 (0–50.0)	14.3 (0–33.3)	0.65	0.36
IIQ-7	23.8 (0–57.1)	4.8 (0–14.3)	<0.01	23.8 (0–57.1)	14.3 (0–33.3)	0.18	0.12
ICIQ	5(0–14)	4(0–13)	0.80	4(0–10)	5(0–9)	0.15	0.65
POPDI	12(5–21)	2.0(0–6)	<0.001	9(4–16)	2(0–5)	<0.001	0.25

* Mann−Whitney U test. Data are given as median (range).

**Table 4:** Urodynamic changes in both groups before and 6 months after surgery. Data are given as N (%) or mean ± standard deviation.

Parameters	Self-Limiting (N = 15)			No Self-Limiting (N = 35)			Intergroup Post-Op
	Pre-Op	Post-Op	*p* Value	Pre-Op	Post-Op	*p* Value	*p* Value *
DO	8(53.3)	6(40)	0.33 *	10(28.6)	6(17.1)	0.21 *	
ISD	2	0		0	0		
Qmax (mL/s)	15.5 ± 10.5	25.0 ± 18.3	0.31 **	16.6 ± 10.3	20.5 ± 7.9	0.14 *	0.89 **
RU (mL)	57(7–273)	14(0–33)	0.02 ^#	90(9–220)	34(0–75)	0.02 ^#	0.06 ^
FS (mL)	161.8 ± 64.5	149.0 ± 47.7	0.61 **	181.5 ± 78.5	141.2 ± 61.8	0.03 *#	0.88 **
MCC (mL)	348.8 ± 97.3	303.0 ± 89.4	0.18 **	413.3 ± 172.6	399.5 ± 149.2	0.42 **	0.07 **
Pdet (cmH_2_O)	41.0 ± 22.6	33.7 ± 14.2	0.47 **	28.1 ± 15.1	19.2 ± 12.1	0.08 *	0.06 **
FUL (mm)	21.5 ± 4.5	23.4 ± 7.7	0.57 *	24.6 ± 6.1	23.5 ± 5.6	0.38 **	0.48 **
MUCP (cmH_2_O)	59.9 ± 33.2	43.5 ± 25.3	0.042 **#	62.0 ± 40.7	52.0 ± 20.1	0.25 **	0.88 **
UCA (mmcmH_2_O)	752.5 ± 477.9	626.3 ± 409.5	0.185 **	801.9 ± 514.2	757.6 ± 410.4	0.52 **	0.20 **

Pre-op, preoperative; Post-op, postoperative; DO, detrusor overactivity; ISD, intrinsic sphincter deficiency, Qmax, maximum flow rate; RU, residual urine; FS, first sensation to void; MCC, maximum cystometric capacity; Pdet, detrusor pressure at peak flow; FUL, functional urethral length; MUCP, maximum urethral closure pressure; UCA, urethral closure area. * Chi-square test; ** Paired *t*-test, ^ Mann−Whitney U test; # Statistical significance.

## Data Availability

The data that support the findings of this study are not publicly available due to patient privacy and ethical restrictions. Data may be available from the corresponding author upon reasonable request and with approval from the Institutional Ethics Committee of Kaohsiung Medical University Hospital.
